# A New Sign Language for Deaf Mutes; Being a Thesis, Etc.

**Published:** 1851-08

**Authors:** Albert J. Myer


*A new Sign Language for Deaf Mutes; being a Thesis, etc.* By ALBERT J. MYER.

The ingenious and interesting thesis by Dr. Myer, published in the June No. of this Journal, has been issued in a neat pamphlet form. We are gratified to see that it meets with a very favorable reception. The plan proposed is novel, and if found to be successful in facilitating intercommunication among the unfortunate class for whose benefit it is designed, will secure for the author an enviable reputation, and, what is of more value, the pleasing consciousness of having rendered valuable service to philanthrophy.

